# Single-cell transcriptome in the identification of disease biomarkers: opportunities and challenges

**DOI:** 10.1186/s12967-014-0212-3

**Published:** 2014-08-12

**Authors:** Zhitu Zhu, Diane C Wang, Laurenţiu M Popescu, Xiangdong Wang

**Affiliations:** Department of Oncology, Liaoning Medical University Hospital, Jinzhou, China; Department of Medicine, Royal Berkshire Hospital, Reading, UK; Department of Morphological Sciences, Division of Cellular and Molecular Medicine, Faculty of Medicine, Carol Davila University of Medicine and Pharmacy, Bucharest, Romania; Fudan University Center of Clinical Bioinformatics, Shanghai Respiratory Research Institute, Fudan University School of Medicine, Zhongshan Hospital, Shanghai, China

**Keywords:** Single cell, Sequencing, Biomarkers, Heterogeneity, Disease

## Abstract

Single cell transcriptome defined as the entire RNA or polyadenylated products of RNA polymerase II on a cell can describe the gene regulation networks responsible for physiological functions, behaviours, and phenotypes in response to signals and microenvironmental changes. Single cell transcriptome/sequencing has the special power to investigate small groups of differentiating cells, circulating tumour cells, or tissue stem cells. A large number of factors may influence the extent of single-cell heterogeneity within a system. It is the opportunity that the single-cell sequencing can be used for the identification of genetic changes in rare cells, e.g. cancer and tissue stem cells, in clinical samples. The methodologies of single-cell sequencing have been improved and developed with the increase of the understanding and attention. The clinical research and application of the single cell sequencing analysis are expected to identify and validate disease-specific biomarkers, network biomarkers, dynamic network biomarkers. The single cell research and value will be also dependent upon the understanding of genomic heterogeneity, planning and design of study protocol, representative of selected and targeted cells, and sensitivity and repeatability of the methodology. The single cell sequencing can be used to develop new diagnostics, monitor disease progresses, measure responses to therapies, and predict the prognosis of patients, although there are still a large number of challenges and difficulties to be faced. It would be more values and specificities of the single cell sequencing to integrate with the function of cells, organs, and systems of the body, the clinical phenotypes of patients, and the description of clinical bioinformatics.

Single cell transcriptome defined as the entire RNA or polyadenylated products of RNA polymerase II on a cell can describe the gene regulation networks responsible for physiological functions, behaviours, and phenotypes in response to signals and microenvironmental changes. Methods of single cell transcriptome analyses have been developed and significantly improved with advantages and disadvantages [1,2]. Single cell transcriptome can demonstrate the heterogeneity of gene expressions, interactions and regulations of gene regulatory networks, subpopulations within a tumour, characteristics of putative cancer stem cells, gene expression profiles of intracellular compartments, mRNA locations, allele specific gene expression, or the information combined with the existing strand-specific cDNA library preparation strategies. With next generation sequencing, the information on both full genome and transcriptome can be obtained from an individual cell. Single-cell genome analysis becomes more important in the understanding of the diversity in microbial ecology, cancer development, prenatal genetic diagnosis, or human genome structures.

Single cell transcriptome/sequencing has the special power to investigate small groups of differentiating cells, circulating tumour cells, or tissue stem cells. Tang et al. defined the dynamic molecular changes with cell-fate changes during the conversion from the inner cell mass cells of blastocysts to pluripotent embryonic stem cells using RNA-Seq transcriptome analysis at the resolution of single cells [3]. Molecular mechanisms by which genetic elements could be switched depend upon the pluripotency, changes in epigenetic regulators, the stability of the newly acquired epigenotype, or self-renewal. It should be an excellent example to further study the regulation and differentiation of small numbers of stem cells in adults, genes for general metabolism, expressions of repressive epigenetic regulators, or changes in microRNAs, to identify targeted genes of pluripotency. Furthermore, single cell transcriptome was used to measure the frequency of large-scale genome instability and de novo mutation rates with distinct characteristics [4].

A large number of factors may influence the extent of single-cell heterogeneity within a system, e.g. the source of analyzed tissues, biological conditions or microenvironments of the individual cell. The single cell transcriptome was suggested to avoid, or at least reduce, the variability of clinical samples, in order to define new cell classifications, transitional states, biological distinctions, or biomarkers. Shalek et al. recently demonstrated that the inflammatory stimulus lipopolysaccharide could induce an extensive, previously unobserved, bimodal variation in mRNA abundance and splicing patterns in bone marrow-derived dendritic cells using single-cell RNA-Seq analysis [5]. It indicates that stimuli from autocrine-based or external sources may initiate the heterogeneity of the cell, different from the traditional understanding. It is possible that we can identify and discover more sensitive and specific elements responsible for the development of cell heterogeneity as biomarkers to monitor dynamic changes and interactions of gene regulation networks or develop new molecular or cellular therapies. The multiplexed primer design can be introduced for selected targeted genes to lower the pre-amplification primer concentration and primer dimer signals, increase the feasibility of measurements, or make the operation easier for classification of cell type, dissection of heterogeneity, mapping of cellular hierarchy, and computational construction of genetic networks [6].

It is the opportunity that the single-cell sequencing can be used for the identification of genetic changes in rare cells, e.g. cancer and tissue stem cells, in clinical samples which may contain hundred cells in body fluids and biopsy with fine-needle. It seems that one of clinical applications of single-cell sequencing should be to understand the genomic profiling of copy number or sequence mutations in rare cells. The challenge in the identification and validation of therapeutic targets is to define the cell heterogeneity, especially to differ between cancer and pre-cancer cells, activated and non-activated inflammatory cells, or functional and non-functional cells, between sampling micro-regions, between cells at various stages, or between responses to therapy. For example, our previous studies on genetic comparison of mouse lung telocytes with mesenchymal stem cells and fibroblasts demonstrated that about 10-20% of genes expressed differently between 5 and 10 day cell culture of lung telocytes per se [7]. It indicates that gene expressions, phenotypes, or functional networks can be varied in the differentiation process or developmental duration. Single cell sequencing has been widely considered as the powerful and helpful tool to identify disease-specific biomarkers to diagnose and monitor the occurrence and progress of the disease, as well as the responses of patients to the treatment. It has been emphasized that disease-specific biomarkers, network biomarkers, and dynamic network biomarkers can monitor and predict the responses to therapies, prognoses of patients, and reoccurrences of the disease, by integrating genomic/proteomic profiles with clinical phenotypes [8–12]. It is a common challenge for those methodologies including single cell sequencing to define the existence of discovered genes before the occurrence of the disease, before clinical diagnosis, or before the application of therapies. It should be also considered whether the same type of the single cell can be collected for measuring the genomic heterogeneity and frequency of resistant clones in the primary disease before and after a single therapy or multiple manipulations, or during the dynamic follow-up in patients. It is highly expected to have the single cell sequencing study with a clear clinical design and definite disease-specific or associated groups, including similar diagnoses, severities, stages, genetic backgrounds, pathogeneses, or therapies. One of critical parts to apply single-cell sequencing for disease biomarkers should be to integrate clinical experience and knowledge with bioinformatics from the single cell sequencing and ensure the understanding of diseases.

The methodologies of single-cell sequencing have been improved and developed with the increase of the understanding and attention. The single cell sequencing is expected to detect the genomic heterogeneity in the primary disease and provide useful information for selecting therapies, monitoring the efficacy and efficiency of the therapy, predicting the potential outcomes of patients, if the disease would be monogenomic one which hardly exists in clinical practice. It is more important to consider the interactions between genes, proteins, cells, organs, as well as the critical role of the tissue microenvironment, when clinical studies are designed for the single cell sequencing analysis. Single-cell sequencing was evidenced to have the high sensitivity in the identification of rare chemoresistant clones in the primary cancer and to investigate questions of whether resistant clones were pre-existing in the primary cancer or appeared in response to therapies [13]. The challenge to translate single cell sequencing analysis from clinical research to clinical application is to standardize the study design and protocol, the study and operation process, data analysis and mining, and the application of clinical bioinformatics. The clinical application of single cell sequencing per se faces the difficulty of problem-solving or question-based study design, and defines the criteria of disease-specific diagnosis, monitoring, or prediction. On the other hand, the single cell sequencing can be used as the tool to screen and identify disease-specific biomarkers which are further developed into a diagnostic panel. It is still difficult to develop the gene panel-based diagnostic kits after the identification, selection, validation, and optimization from the single cell sequencing, due to the unexpected barriers, e.g. for stability and repeatability of the expression, the representative of the targeted panel, or description of the measured results. It is also important to understand the dynamic existence of genomic variation, interaction between genes, and definition of gene scale, origin, rate, and nature.

Another potential or new hope is to develop new type cells for therapies by bioengineering or modifying targeted genes selected from the single cell sequencing. It believes that the construction of a phylogenetic tree of a human lifetime and the contribution of genetic heterogeneity to the organism can be defined and figured out by the single cell sequencing analysis of the different cells from endodermal, mesodermal, and ectodermal tissues from an individual. The sub-classification or new discovered populations of cell types can be found by gene expression profiles and epigenetic status, although the relationship between genome sequence, epigenetic status, and gene expression, and the functional capacity of the cell remain unclear [14]. The new methods have been suggested to expedite applications in basic genome research and provide novel approaches for clinical genetic diagnosis [15]. The paired-end sequence analysis of single-cell whole-genome amplification products was developed to explore the nature and pace of genome mutation, the genetic composition, or de novo mutations. Such exciting approach could detect DNA mutations, DNA copy number changes from allelic whole-genome amplification -amplification, and the break points and architecture of structural variants. We call the special attention and believe it is a time to further translate the concept of single-cell analysis, sequencing, or transcriptomics into clinical research and application for the identification and validation of disease-special biomarkers, on basis of recent emphases from a growing number of outstanding publications [16–19].

In conclusion, the clinical research and application of the single cell sequencing analysis are expected to identify and validate disease-specific biomarkers, network biomarkers, dynamic network biomarkers. The single cell research and value will be also dependent upon the understanding of genomic heterogeneity, planning and design of study protocol, representative of selected and targeted cells, and sensitivity and repeatability of the methodology. The single cell sequencing can be used develop new diagnostics, monitor disease progresses, measure responses to therapies, and predict the prognosis of patients, although there are still a large number of challenges and difficulties to be faced. It would be more values and specificities of the single cell sequencing to integrate with the function of cells, organs, and systems of the body, the clinical phenotypes of patients, and the description of clinical bioinformatics.
